# A Case of Lance-Adams Syndrome with Mixed Cortical and Reticular Reflex Myoclonus

**DOI:** 10.5334/tohm.684

**Published:** 2022-03-18

**Authors:** Andy Ho Wing Chan, Jamie Nichols, Winona Tse, Sophia L. Ryan

**Affiliations:** 1 Department of Neurology, Mount Sinai Hospital, New York, US

**Keywords:** Myoclonus, Lance-Adams Syndrome, post-hypoxic myoclonus, reticular reflex mycolonus

## Abstract

**Background:**

Lance Adams syndrome is a chronic post-hypoxic myoclonus.

**Phenomenology Shown:**

This video abstract illustrates Lance Adams Syndrome with mixed cortical and reticular reflex myoclonus in a 32-year-old woman following respiratory arrest in the setting of an asthma attack, as well as improvement in her exam following pharmacologic management.

**Educational Value:**

Lance Adams syndrome can include both cortical and reticular reflex myoclonus features while interdisciplinary intervention and pharmacological treatment can improve symptomatology.

A 32-year-old right-handed woman with asthma presented to the hospital with asthma attack complicated by pulseless electrical activity. Return of spontaneous circulation was achieved within 5 minutes and she underwent targeted temperature management for 48 hours. The patient was transferred to our hospital for EEG monitoring. EEG did not show any epileptiform activity or seizures. The patient emerged from a 9-day coma with an apparently normal neurologic exam. Three days later, she collapsed when attempting to stand. The neurologic exam was normal but for myoclonus. Workup included laboratory studies, and MRI, all of which were normal.

As seen in ***Video 1***, (“Before Treatment”), the myoclonus occurred at rest and had an action component. Intermittent bursts of irregular myoclonic jerks were observed in both proximal and distal leg muscles at rest and in arm muscles with the action of holding the arms outstretched. The myoclonus affected distal limbs initially then later involved proximal limbs and both proximal and distal limbs seemed equally affected. Patient was sensitive to tactile stimuli (e.g. examiner touch, physical therapy) but not to visual, auditory nor other stimuli. The myoclonus was more pronounced with action. The patient was diagnosed with chronic post-hypoxic myoclonus [1,2]. Notably, this patient’s myoclonus has some features of reticular reflex myoclonus, including stimulus sensitivity and presence at rest [2].

**Video 1:** **Mixed myoclonus.** This video demonstrated our patient’s mixed cortical and reticular reflex myoclonus prior to treatment and after the treatment (clonazepam and levetiracetam).

Our patient experienced symptomatic improvement with clonazepam 0.5 mg three times a day and levetiracetam 1000 mg four times a day [2]. ***Video 1*** (“After Treatment”) demonstrates decreased frequency and amplitude of the myoclonus. She was thus able to participate in targeted physical therapy and completed a four-week acute rehabilitation program before being discharged home.

On follow up in clinic six months later, she reported infrequent episodes of tactile stimulus-induced myoclonus only, particularly when encountering an unexpected stimulus. There was no myoclonus noted on exam and the patient’s neurologic exam was otherwise normal.

Different types of myoclonus are extremely difficult to differentiate without neurophysiologic testing, which was not available in this case. However, this video abstract demonstrated that Lance Adams syndrome, typically described as a cortical myoclonus, may involve more than one type of myoclonus and different types of myoclonus can coexist in an individual [3]. In this case, our patient has myoclonus with clinical features of both cortical and reticular reflex myoclonus, as well as improvement after physical therapy, pharmacological therapy including clonazepam and levetiracetam and likely time.
